# DNA methylation subgroups in melanoma are associated with proliferative and immunological processes

**DOI:** 10.1186/s12920-015-0147-4

**Published:** 2015-11-06

**Authors:** Martin Lauss, Markus Ringnér, Anna Karlsson, Katja Harbst, Christian Busch, Jürgen Geisler, Per Eystein Lønning, Johan Staaf, Göran Jönsson

**Affiliations:** Department of Oncology and Pathology, Clinical Sciences, Lund University Hospital, Lund University, Lund, 221 85 Sweden; Section of Oncology, Department of Clinical Science, University of Bergen, Bergen, Norway; Department of Clinical Oncology, Haukeland University Hospital, Bergen, Norway; Present Address: Department of Clinical Molecular Biology and Laboratory Sciences, Akershus University Hospital, Lørenskog, Norway; Institute of Clinical Medicine, University of Oslo, Oslo, Norway

**Keywords:** Melanoma, DNA methylation, Gene expression, Melanocytes, Molecular subtypes, Polycomb

## Abstract

**Background:**

DNA methylation at CpG dinucleotides is modified in tumorigenesis with potential impact on transcriptional activity.

**Methods:**

We used the Illumina 450 K platform to evaluate DNA methylation patterns of 50 metastatic melanoma tumors, with matched gene expression data.

**Results:**

We identified three different methylation groups and validated the groups in independent data from The Cancer Genome Atlas. One group displayed hypermethylation of a developmental promoter set, genome-wide demethylation, increased proliferation and activity of the SWI/SNF complex. A second group had a methylation pattern resembling stromal and leukocyte cells, over-expressed an immune signature and had improved survival rates in metastatic tumors (*p* < 0.05). A third group had intermediate methylation levels and expressed both proliferative and immune signatures. The methylation groups corresponded to some degree with previously identified gene expression phenotypes.

**Conclusions:**

Melanoma consists of divergent methylation groups that are distinguished by promoter methylation, proliferation and content of immunological cells.

**Electronic supplementary material:**

The online version of this article (doi:10.1186/s12920-015-0147-4) contains supplementary material, which is available to authorized users.

## Background

The tumor phenotype is formed by genetic and epigenetic events and signals from the micro-environment. In melanoma, the landscape of somatic mutations and gene copy number changes has been explored [1–3], and the understanding of surrounding immunological cells has led to the development of new treatment options [4, 5]. Recurrently mutated genes discovered by recent exome sequencing studies are frequently involved in epigenetic modifications [6], e.g., *ARID2* [1, 2], pointing to a yet under-appreciated driver role of epigenetic factors. Methylation of cytosine in the CpG context is an epigenetic mark that can directly influence transcriptional activity, and changes of DNA methylation pattern have been associated with tumorigenesis. In melanoma, a number of genes were found to harbor promoter CpG island methylation [7]. Consequently, melanomas hypermethylated at several CpG islands were termed CpG Island Methylator Phenotype (CIMP) [8], according to findings from other cancer types [9, 10]. Promoter methylation is reported to repress gene expression. Conversely, a small number of genes, such as *MAGE*, are activated by hypomethylation in melanoma [7]. High-throughput technology has enabled the assessment of DNA methylation of a wider spectrum of genomic locations in melanoma [11–23], often highlighting methylation events that discriminate melanoma from nevi/melanocytes. Genome-wide methylation patterns may reveal tumor subtypes of biological and clinical relevance. In this respect, Thomas et al. used the Illumina GoldenGate platform (1505 CpGs) and found three methylation subgroups in 47 primary cutaneous melanoma, with the hypermethylated groups having increased Breslow thickness [24]. Sigalotti et al. analyzed short-term cultures from 45 stage IIIC patients on Illumina 27 K BeadChips and identified one group with low and one with high average methylation, respectively; with the latter group having poor overall survival [25].

Previously, we introduced four gene expression subgroups in melanoma; one proliferative group (‘Proliferative’), one group with activated melanogenesis pathway (‘Pigmentation’), one group displaying elevated immune response (‘High-Immune’) and one with contribution from surrounding normal tissue (‘Normal-like’) [26]. The groups were also present in primary melanomas and had diverging clinical outcome [27]. In this study we aimed to characterize DNA methylation subtypes of melanoma, validate the findings in independent data, and to investigate the relationship of methylation and gene expression patterns.

## Results

### Identification of three methylation subtypes in melanoma

**Fig. 1:**
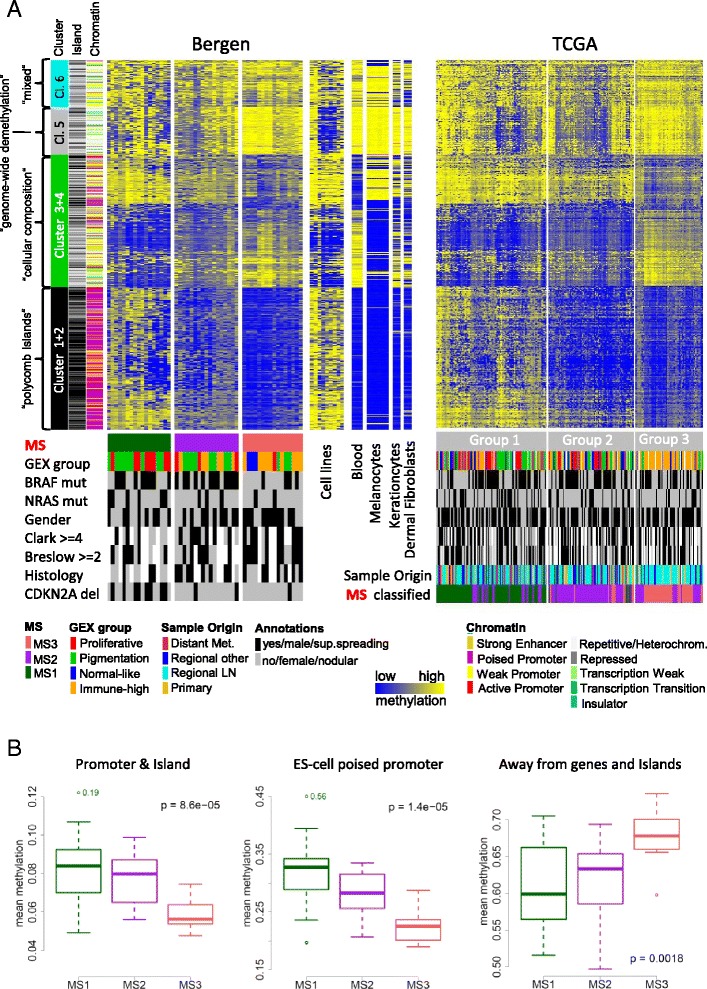
Three melanoma methylation subtypes. **a** Methylation lanes from left to right: 3 methylation subtypes (MS) from Bergen tumors, 9 cell lines, blood leucocytes, melanocytes (two lanes of each light, medium and dark melanocytes), dermal epidermis, dermal fibroblasts; and 3 unsupervised groups from TCGA tumors. Beta values in heatmap are from unmethylated, blue to methylated, yellow. *Cluster* lane displays the four main CpG probe clusters of the 9,886 melanoma-methylated and 5,236 melanoma-demethylated CpGs, using unsupervised hierarchical clustering in the Bergen data. *Island* lane: black = CpG island, dark grey = shore/shelf, light grey = open sea. *Chromatin* lane refers to embryonic stem cell chromatin states. GEX = gene expression. CDKN2Adel = homozygous deletion of the *CDKN2A* locus. **b** Mean beta value across MS subtypes in different genome-wide CpG sets. Promoter & Island = annotated as TSS200 or TSS1500 (i.e. up to 1500 bp from transcription start site) and annotated as CpG Island, *n* = 57,579 CpGs. Away from genes and Island = no annotation for gene and island, *n* = 42,728 CpGs. Poised promoter = embryonic stem cell chromatin state, see panel A, *n* = 59,901 CpGs. *P*-value from Kruskal-Wallis test

We determined the methylation status of more than 480,000 individual CpGs in 50 metastatic melanoma tumors, subsequently called ‘Bergen’ data, that were included in a previous discovery of gene expression phenotypes [26] (Table 1). The methylation measurements are β-values that range from 0, unmethylated to 1, fully methylated [28]. We defined a set of CpGs that is aberrantly methylated in melanoma as compared to melanocytes, with 9,886 melanoma-methylated CpGs and 5,236 melanoma-demethylated CpGs, respectively, as previously described [29]. Unsupervised clustering of the combined CpG sets identified three consensus groups in the Bergen melanomas, subsequently called methylation subtype 1, 2 and 3 (MS1, MS2, MS3) (Fig. 1a). As validation data, we used 242 samples from The Cancer Genome Atlas (TCGA) (Table 1). Unsupervised clustering of the TCGA data, which consists of primary and metastatic tumors, using the same CpG set, resulted in three groups with comparable methylation patterns to the Bergen data (Fig. 1a). Principal component analyses were performed to exclude that the groups are due to technical procedures at data generation, such as the use of different batches (Additional file 1: Figure S1A and B). The three groups were re-identified in both datasets when changing the number of CpGs to be included in the clustering procedure (Additional file 1: Figure S1C). We next classified the TCGA samples into MS1, MS2, and MS3 groups using Bergen-derived methylation centroids (Additional file 2: Table S1). Centroid classification was the dominant explainer of total variation in TCGA methylation data compared to histopathological and molecular factors (Additional file 1: Figure S1B). Furthermore, classification of TCGA tumors using Bergen-derived centroids, is in good agreement with the groups obtained from unsupervised clustering of the TCGA data (76 % sample co-occurence, *P* < 2x10^−16^, Additional file 1: Figure S1D and “MS classified” annotation in Fig. 1a), confirming that the methylation clusters of Bergen are re-identified in the TCGA data.

**Table 1:** Patient and sample information

Feature	Bergen (*n* = 50)	TCGA (*n* = 242)
*Gender*		
Male	28 (56)	154 (64)
Female	22 (44)	88 (36)
Median thickness (mm)	2.25 (0.5-25)	2.7 (0.28-50)
Clark		
I	3 (9)	3 (2)
II	2 (6)	13 (7)
III	9 (27)	49 (27)
IV	13 (39)	83 (46)
V	6 (18)	31 (17)
*Histogroup*		
SSM	18 (53)	-
NM	16 (47)	-
*Sample origin*		
Primary	0	27 (11)
Regional LN	3 (6)	134 (56)
Regional other	0	49 (20)
Distant metastasis	47 (94)	31 (13)
*BRAF mutation*		
*yes*	25 (50)	118 (49)
*no*	25 (50)	123 (51)
*NRAS mutation*		
*yes*	11 (22)	66 (27)
*no*	39 (78)	175 (73)
*CDKN2A-arrayCGH*		
Homozygous Deletion	11 (22)	-
Present	39 (78)	-
*Gene expression phenotype*		
High-immune	15 (30)	69 (32)
Normal-like	3 (6)	33 (15)
Pigmentation	20 (40)	63 (29)
Proliferative	12 (24)	49 (23)

We then restricted the analysis to a subset of CpGs located in CpG islands within 1500 bp upstream of transcription start sites. The vast majority of these promoter island CpGs were hyper-methylated in melanomas (930 of 947), in line with other tumor types. In both datasets, methylation levels spanned a continuous gradient from unmethylated melanomas to fully methylated melanomas, rather than forming a distinctive CIMP group (Additional file 1: Figure S2). We conclude that in melanomas the full range of CpGs provides additional information to the traditionally studied promoter island CpGs, and allowed for the identification of three reproducible methylation subtypes, MS1/2/3.

### Biological processes shaping the methylation subtypes

Methylation levels at promoter islands are elevated in MS1 tumors in comparison to MS3 tumors (Fig. 1b, Kruskal-Wallis test). ‘Poised promoters’ are characterized by Polycomb-complex 2 (PRC2) induced H3K27 tri-methylation marks in addition to H3K4 tri-methylation marks, and are most prevalent in embryonic stem cells [30]. The poised promoter set of embryonic stem cells is important in developmental processes and found to be methylated in various cancer types [31, 32]. Notably, CpGs in this poised promoter set also displayed increased methylation levels in the MS1 group. Conversely, intergenic methylation levels away from genes and islands are reduced in MS1 tumors (Fig. 1b, Kruskal-Wallis test). The MS1 group showed a pattern similar to melanocytes but with hyper-methylation at many poised promoters. The nine melanoma cell lines also displayed the MS1 pattern (Fig. 1a). In contrast, the DNA methylation pattern of the MS3 group was similar to peripheral blood leukocytes. Finally, the MS2 group presents a mixture of MS1 and MS3 methylation patterns with intermediate methylation levels for the majority of CpGs.

**Fig. 2:**
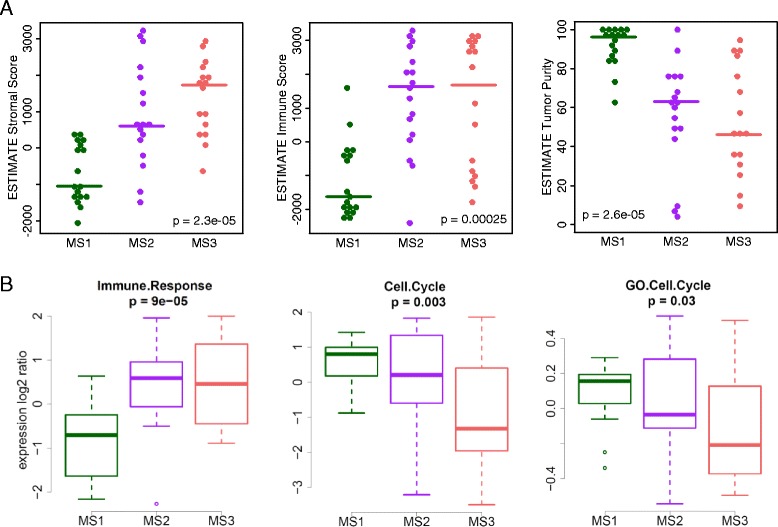
Immunological and proliferative signature expression across methylation subtypes. **a** ESTIMATE scores and tumor purity. *P*-value from Kruskal-Wallis test. **b** Mean expression values of melanoma gene modules and GO-term ‘cell cycle’. *P*-value from anova. Bergen tumor data

The CpG set used for group discovery consisted of four functional clusters (Fig. 1a). First, one CpG cluster (Fig. 1a, black cluster “Cl. 1 + 2”) was highly enriched in the aforementioned Polycomb-targeted poised promoters. This CpG cluster was hypermethylated in melanoma compared to melanocytes with gradually increasing methylation levels from MS3 to MS1 tumors. Second, genome-wide hypomethylation was observed in repetitive, heterochromatic regions, that had gradually decreasing methylation levels from MS3 to MS1 tumors (Fig. 1a, grey cluster “Cl. 5”). Third, a heterogeneous CpG cluster was found that had opposite methylation status in melanocytes and blood leukocytes, with melanoma samples spanning the gradient in between (Fig. 1a, green cluster “Cl. 3 + 4”). The fourth cluster of CpGs could not be assigned to a biological function (Fig. 1a, ‘mixed’, pale blue cluster “Cl. 6”). As the third cluster seemed to contain methylation signals from non-tumoral cells, we used the ESTIMATE algorithm to further investigate the cellular composition of the tumors [33]. Evidence of elevated stromal and immune cell content was observed in MS2/3 compared to MS1 samples (Fig. 2a, Kruskal-Wallis test). Overall, tumor purity was estimated to be lowest in MS3 samples and highest in MS1 samples (Fig. 2a, Kruskal-Wallis test). Furthermore, MS1 and MS2 over-expressed mitotic gene signatures, whereas MS2 and MS3 over-expressed an immune response signature (Fig. 2b, ANOVA). These observations were validated in TCGA data (Additional file 1: Figure S3). In conclusion, the molecular subtypes are shaped by various degrees of promoter hypermethylation, genome-wide hypomethylation, and cell-type specific methylation signatures from the micro-environment.

### Methylation processes of melanoma in comparison to other cancer types

To set the methylation processes of melanoma in context to other cancer types, we used TCGA data from colon cancer (295 tumors, 38 normal tissues), breast cancer (201 tumors, 40 normal tissues) and lung cancer (lung adenocarcinoma, 452 tumors, 32 normal tissues). For these cancer types, we derived tumor-methylated and tumor-demethylated CpGs in the same way as for our melanoma data. The majority of methylated and demethylated CpGs occurred in only one cancer-type (Additional file 1: Figure S4A). Yet, at the gene level the agreement across cancer types was larger, particularly the agreement of methylated genes was considerable (Additional file 1: Figure S4A). Importantly, for all investigated cancer types, the methylated CpGs were located within poised promoters (i.e. PRC2 target genes), and the demethylated CpGs located within heterochromatic regions of ES cells chromatin (Additional file 1: Figure S4B). Similarly, for melanocyte chromatin states, methylated CpGs were situated in bivalently marked chromatin regions, and demethylated CpGs in quiescent/repressed regions (Additional file 1: Figure S4C). In an aim to find melanoma-specific mechanisms of methylation, we compared genes hypermethylated exclusively in melanoma to genes hypermethylated in colon, lung and breast cancer. Genes exclusively methylated in melanoma were significantly enriched for the GO-terms “catalytic activity”, “nucleoside-triphosphatase regulator activity” and “GTPase regulator activity” (false discovery rate (fdr) < 5 %). This may allow speculating that a metabolic process exists in melanocytes that is connected to the MAPK pathway via regulation of GTPase (e.g., Ras) activity, with methylation of the regulators emerging at tumorigenesis. In summary, aberrant methylation in tumors preferentially occurs at genes with “poised promoters” and demethylation in heterochromatin, however, the exact stretches of DNA that get aberrantly methylated are cancer-type specific. This resembles findings from Sproul et al., who however had not included melanomas in their study [34].

### Correlation to histopathology, mutation data and clinical outcome

**Fig. 3:**
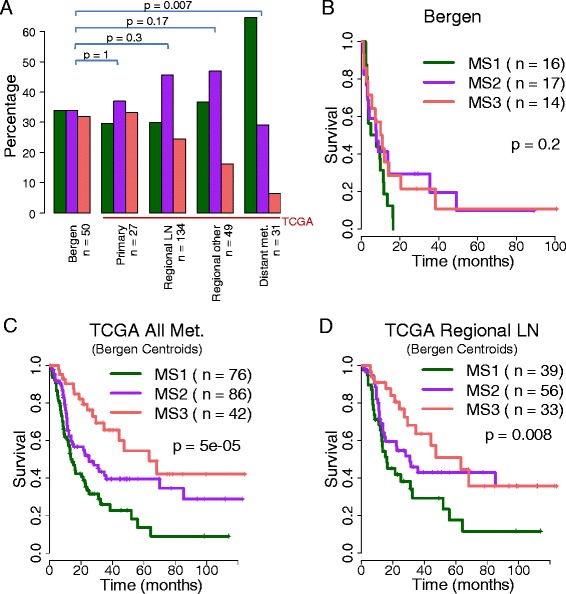
Clinical evaluation of methylation subtypes. **a** Distribution of methylation subtypes in Bergen and TCGA cohorts. *P*-values from Fisher’s exact test. **b**, **c**, **d** Survival analyses. *P*-values from Cox regression

Overall the proportions of the three methylation groups were comparable between the Bergen and TCGA samples. However, the TCGA distant metastasis samples contained more MS1 samples than the Bergen cohort (Fig. 3a, Fisher’s exact test). We did not observe any significant associations of gender, age, primary tumor features (Breslow thickness, Clark’s level, SSM/NM), LDH, lymph-node/subcutaneous, CD3 or CD20 IHC with the methylation groups in the Bergen data. Furthermore, in our data, neither *BRAF* nor *NRAS* mutation status was clearly associated with either subgroup (Fig. 1a). Homozygous deletions of the *CDKN2A* locus seemed to be more frequent in MS1 (*p* = 0.02). We repeated the mutation analysis for TCGA exome-sequencing data. The total number of mutations in tumors was equally distributed across subtypes (Additional file 1: Figure S5A). For mutations in 15 known melanoma driver genes [1, 2] we did not observe any subtype association, with an fdr < 10 % (Additional file 1: Figure S5B). *IDH1* hotspot mutations at R132 correlate with a glioblastoma hypermethylation phenotype [35] and have been reported in melanoma [36]. *IDH1* hotspot mutation was more prevalent in the hypermethylated MS1 group (6 of 11 mutated cases), however, not reaching significance.

Disease-specific survival analysis indicated more aggressive behavior of MS1 melanomas compared to MS2/3 melanomas, although not reaching significance (*p* = 0.2 log-rank test, Fig. 3b). This trend was further supported in metastatic TCGA tumors, with MS1 patients having inferior survival outcome (*p* = 5x10^−5^, Fig. 3c). As samples from distant metastases are over-represented in the MS1 subtype, we repeated the survival analysis using only the regional lymph node metastasis from TCGA, and again observed inferior survival of MS1 patients (*p* = 0.008, Fig. 3d). The primary TCGA cohort size (*n* = 27) was too small to allow for survival analysis.

### A link between methylation and gene expression phenotypes

**Fig. 4:**
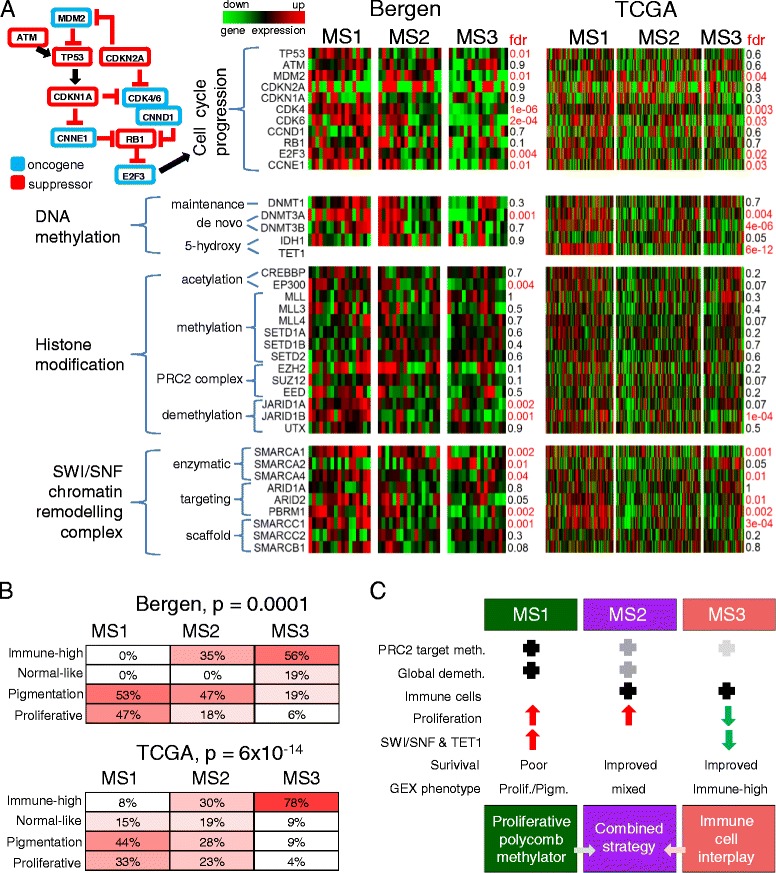
Association between transcription and methylation landscapes. **a** Expression of genes involved in cancer-driving pathways and processes, across methylation subtypes. fdr = false discovery rate for *p*-values from anova using Benjamini-Hochberg adjustment. **b** Heatmap of association between gene expression and methylation subtypes. *P*-values from Fisher’s exact test. **c** Summary cartoon. Check sign = presence, up-arrow = up-regulation, down-arrow = down-regulation. MS = Methylation subtype. GEX = Gene expression. Prolif. = Proliferative subtype. Pigm. = Pigmentation subtype

The MAPK and PI(3)K pathways are instrumental in melanoma [37, 38]. However, these pathways did not seem to be differentially expressed across methylation subtypes in Bergen and TCGA data, with the exception of *DUSP* genes (Additional file 1: Figure S6A). Genes that promote the cell cycle, such as *MDM2*, *CDK4*, *CDK6*, *CCND1*, *CCNE1* and *E2F3* are up-regulated in MS1 samples; as well as genes that directly modify CpG methylation, such as *de-novo* methyltransferase *DNMT3A* and 5-hydroxy converting *TET1* (fdr < 5 %, Fig. 4a, ANOVA with Benjamini-Hochberg fdr). Histone modifiers were generally not differentially expressed across methylation subtypes, again with an exception, the H3K4 de-methylation enzyme *JARID1B*. Recently, members of the SWI/SNF chromatin remodeling complex have been found to carry loss-of-function mutations across cancer types, implying a tumor suppressor role [39, 40]. Interestingly, several SWI/SNF members are up-regulated in MS1 tumors (Fig. 4a).

To further investigate the relationship of methylation to gene expression patterns, we compared the methylation subtypes to the four gene expression phenotypes previously defined in the Bergen cohort [26]. MS1 consisted of ‘Proliferative’ and ‘Pigmentation’–type tumors (Fig. 4b), suggesting a link between proliferation and hyper-methylation. The MS3 subgroup is dominated by ‘High-immune’ and ‘Normal-like’ tumors. The agreement of gene expression subtypes and methylation subtypes is statistically significant (*p* = 0.0001, Fisher’s exact test, Cross-table Additional file 1: Figure S6B), with methylation subtypes providing additional stratification beyond gene expression subtypes. The co-occurrence of methylation and gene expression phenotypes was also observed in the TCGA data (*p* = 6x10^−14^, Fisher’s exact test, Fig. 4b and Additional file 1: Figure S6B). Together, these findings provide a link between the transcriptional and DNA methylation landscapes in melanoma.

## Discussion

In the present study, we have identified three groups of melanoma specimens with different methylation patterns. The MS1 group fits the paradigm of genome-wide de-methylation and focal promoter hyper-methylation in tumors [41]. Most cancer types studied by the TCGA consortium, contained a subgroup with high methylation levels [42]. Hypermethylated subgroups in colon and endometrial cancer were associated with *MLH1* inactivation and increased mutation rate. In other cancer types hypermethylated subgroups were associated with *IDH1* mutations (AML, glioblastoma), or EBV virus (gastric cancer). For breast cancer, lung squamous cell carcinoma and lung adenocarcinoma, there has been no clear association of the methylated group to such a factor. Likewise, our melanoma hypermethylated group MS1 was not significantly associated with a mutated gene or overall mutation rate. Hyper-methylation is primarily directed towards the embryonic PRC2 target gene set. These genes are pre-marked by histone H3K27 tri-methylation in addition to a H3K4 mark in embryonic stem cells [43]. DNA methylation of these ‘poised promoters’ has emerged as a general process across tumor types [31, 44]. Genome-wide de-methylation in melanoma occurs in repetitive regions, distant from CpG islands. A relatively small number of de-methylated CpGs are captured on the array, which is likely due to difficulties with designing probes in repetitive regions.Cell line methylation patterns differed from normal melanocytes and matched well with the MS1 group. Moreover, methylation subtypes seemed to be influenced by the cellular composition of the tumor, in particular, the MS3 methylation pattern is closely related to blood leukocytes and has a reduced tumor cell content compared to MS1. In the future, single-cell sequencing may clarify the methylation pattern of pure MS3 tumor cells. However, for prediction of immune therapy success, information from both tumor and surrounding cells has been valuable [45–48]. Therefore, methylation signals from the micro-environment may prove useful for decision making in melanoma.

Together, the methylation data reveals two tumor strategies (Fig. 4c). First, a proliferative strategy that co-occurs with hypermethylation of poised promoters. Poised promoters are located in differentiation genes, some of them potentially negative for tumor fitness. It is therefore tempting to speculate that methylation of poised promoters is aimed at removing the brakes of tumor proliferation. Sequential methylation of the poised promoter set may inactivate repressive developmental genes, resulting in a small advantage in tumor growth. Therefore, clones with ever-increasing poised promoter methylation may be preferred in tumor evolution. Accordingly, instead of a clear CIMP group, we observe a gradual increase in promoter methylation. This proliferation strategy also seems to act independently from the MAPK and PI(3)K signaling pathways (Additional file 1: Figure S6A). The second strategy is based on an interplay of tumor cells with immune cells. The composition of the immune cells in the melanoma samples was preserved across cohorts, as the methylation subtypes could be validated in the independent TCGA data. The precise interplay of tumor and immune cells is still unclear. The knowledge of this interplay may reveal the basis of improved survival of MS3 patients, and could greatly benefit the development of further immune therapies. Importantly, immunomodulating agents have demonstrated significant clinical benefit in melanoma and include immune checkpoint blockade agents such as anti-CTLA4 and anti-PD1/PDL1 [4, 5]. In contrast, tumor-associated macrophages, regulatory T-cells and myeloid-derived suppressor cells can blunt anti-tumor effector functions and promote tumor growth and invasiveness, highlighting the complexity of the interplay between the tumor and the immune system.

A major limitation of the current study is that DNA methylation is the only investigated epigenetic mark. Characterization of a broad spectrum of epigenetic marks will most likely enhance insight into melanoma. In addition, a better understanding of the functional role of the SWI/SNF chromatin remodeling complex and its impact on the epigenetic landscape will be very useful.

## Conclusions

Herein, we identified three methylation subgroups in melanoma, with distinct biological properties and disease outcome. The methylation groups are in good agreement with gene expression phenotypes, and highlight two different mechanisms underlying this disease, a proliferative strategy and a strategy based on the presence of immune cells.

## Methods

### Generation of genome-wide methylation data

We determined genome-wide methylation levels in 50 tumors from Haukeland University Hospital in Bergen [26], 9 melanoma cell lines (A2058, A7, CHL1, HT144, MM383, SKMEL3, SKMEL5, WM239A, WM852), light, medium, dark melanocytes, dermal epidermis and fibroblasts (Science Cell Research, USA), and peripheral blood leukocytes (Promega), using Illumina Infinium HumanMethylation450K BeadChips. Informed consent was obtained from all patients. The study was approved by the local ethics committee. DNA was extracted as described previously [26]. Bisulfite conversion and hybridization to Infinium HumanMethylation450K BeadChips was performed following the manufacturer’s instructions, with samples being processed as one batch. Methylation data had been deposited at Gene Expression Omnibus as series GSE51547, and gene expression data as GSE22153.

### Processing of Bergen methylation and gene expression data

Beta-values were calculated from methylated (M) and unmethylated (U) signal, as beta = M/(M + U). A total of 496 missing values were imputed using k-nearest neighbor imputation (k = 10) [49]. For each sample we performed a peak-based correction of Illumina I and II chemical assays similar to Dedeurwaerder et al. [50]. For each assay the beta values were smoothened (Epanechnikov kernel) to estimate unmethylated and methylated peaks, respectively. The unmethylated peak was moved to 0 and the methylated peak to 1 using linear scaling, with beta-values in between stretched accordingly and capped at 0 and 1. CpGs of chromosomes X and Y were removed.

The matched gene expression data was processed as described previously [26]. Multiple probes for a gene (based on gene symbol) were median-merged.

### Processing of TCGA methylation and gene expression data

Methylation data for 242 samples from the TCGA data portal https://tcga-data.nci.nih.gov/tcga/ were processed as described for the Bergen data. For a subset of 214 samples we downloaded normalized RSEM gene counts from ‘level 3’ RNA sequencing data. The data was quantile-normalized using *limma* [51], added an offset of 32, capped to a maximum value of 65,000, log_2_ transformed, and genes were median-centered. The samples were classified using reported gene expression phenotype centroids as described [26]. TCGA methylation data for colon cancer (COAD, 295 tumors, 38 solid normal tissues), breast cancer (BRCA, 201 tumors, 40 solid normal tissues, constituting the first six TCGA batches) and lung adenocarcinoma (LUAD, 452 tumors, 32 solid normal tissues) were processed as described for the Bergen data.

### Clustering procedure to obtain consensus clusters

The set of 9,886 melanoma-methylated CpGs was defined as β < 0.1 in melanocytes and β > 0.5 in at least 20 % of tumors (*n* = 10). The set of 5,236 melanoma-demethylated CpGs was defined as β > 0.9 in melanocytes and β < 0.5 in at least 20 % of tumors (*n* = 10), respectively. The CpG sets were combined for the clustering procedure. Hierarchical Clustering Analysis (HCA) with Euclidean distance and Ward’s algorithm for agglomeration, was performed for 1000 bootstrapped versions of the data [44]. At each bootstrap HCA the dendrogram is cut into either 2 or 3 clusters. For all sample pairs, the frequency with which the two samples have clustered into the same group is calculated. The co-clustering frequency matrix is then reordered by HCA (Pearson correlation distance, Ward agglomeration) to obtain the consensus methylation groups, i.e. subsets of samples that repeatedly cluster together.

### Nearest Centroid Classification

The centroids of the methylation subgroups were defined as the mean methylation values of the 9,886 melanoma-methylated and 5,236 melanoma-demethylated CpGs, across each subgroup of the Bergen samples. Therefore, we obtained three centroids, one for MS1, one for MS2, and one for MS3, presenting the mean beta-values of the subgroups. The centroids were deposited as Additional file 2: Table S1. To classify a sample from the TCGA HumanMethylation450K data, the beta-values of the centroid CpGs were extracted. The Euclidean distance of the TCGA beta-values to each of the three centroids was calculated and the sample was assigned to the MS subgroup to whose centroid it had the shortest distance. Using this classification system we assigned each TCGA tumor as being either MS1, MS2 or MS3.

### Statistical analysis

We used chromatin predictions for H1 embryonic stem cells [52], and melanoma gene co-expression modules for ‘Immune Response’ and ‘Cell Cycle’ from an independent cohort [53]. All statistical analysis was performed in R 3.1.1. Survival analysis was performed using the *survival* package, principal component analysis was performed using the *swamp* package [54], ESTIMATE scores were derived using the *estimate* package [33].

## Additional files

Additional file 1: Figure S1. Robustness of methylation subtypes. Principal component analysis to monitor data bias due to technical variables in the Bergen (**A**) and TCGA (**B**) cohorts, respectively. The entire set of 473,864 CpGs was used for principal component analysis. The heatmaps indicate the association of a sample annotation to each of the principal components. The strength of association is specified by the log_10_ *p*-value of the linear model with the respective principal component as dependent variable and sample annotation as regressor. DNAconc = DNA concentration. Technical variables of TCGA data are termed as defined by the TCGA consortium. TCGA = The Cancer Genome Atlas Consortium. Abbreviations as in Figure 1 of the main manuscript. (**C**) Sample overlap when using different CpG sets for group discovery in Bergen and TCGA data, respectively. For group discovery we used CpGs with variant methylation between melanoma and melanocytes. CpGs were required to be either methylated or de-methylated in at least 10 tumors for our final subtypes. The CpG set for 10 tumors contained 9,886 melanoma-methylated and 5,236 melanoma-demethylated CpGs. The heatmap displays the overlap of the resulting three consensus clusters at various tumor cutoffs. (**D**) Heatmap of TCGA sample co-occurrence between unsupervised consensus clusters and methylation subtypes obtained from (supervised) nearest centroid classification. **Figure S2.** Promoter island consensus clusters. The group discovery CpG set was reduced to CpGs located within 1500 bp upstream of transcription start sites and within a CpG island. In total this analysis includes 947 CpGs, of which 930 are hypermethylated, and 17 are hypomethylated in tumors as compared to melanocytes. Two-group consensus solutions were favorable to three-group solutions in Bergen and TCGA data, respectively. **Figure S3.** Signature expression across methylation subtypes in TCGA data. (**A**) ESTIMATE scores and tumor purity. *P*-value from Kruskal-Wallis test. (**B**) Mean expression values of gene modules and GO-term ‘cell cycle’. *P*-value from anova. TCGA tumor data. **Figure S4.** Aberrant methylation in melanoma compared to colon, lung and breast cancer. (**A**) Venn diagrams of aberrant methylation on the CpG and gene level. (**B**) Aberrant methylation in ES-cell chromatin context. Chromatin categories as in ENCODE project. (**C**) Aberrant methylation in melanocyte chromatin context. Chromatin categories as in Epigenome Roadmap project. **Figure S5.** Driver gene mutations and methylation subtypes in the TCGA cohort. (**A**) Number of non-silent mutations per sample across methylation subtypes. (**B**) Non-silent and hotspot mutations of reported melanoma driver genes across subtypes. **Figure S6.** Expression of MAPK and PI(3)K pathway genes across methylation subtypes. (**A**) Expression of MAPK and PI(3)K pathway. fdr = false discovery rate for *p*-values from anova using Benjamini-Hochberg adjustment. (**B**) Cross-table of gene expression and methylation subtypes. (PDF 865 kb) Additional file 2: Table S1. Centroids of methylation subgroups. (XLS 1550 kb)

## Abbreviations

CIMP: CpG island methylator phenotype
TCGA: The cancer genome atlas
HCA: Hierarchical clustering analysis
MS: Methylation subtype
NM: Nodular melanoma
SSM: Superficial spreading melanoma
LDH: Lactate dehydrogenase
IHC: Immunohistochemistry
SWI/SNF: SWItch/Sucrose nonfermentable
PRC2: Polycomb repressive complex 2
