# Supplementary material for: DNA methylation subgroups in melanoma are associated with proliferative and immunological processes
Source: BMC Med Genomics. 2015 Nov 6;8:73. doi: 10.1186/s12920-015-0147-4 (PMC4636848; doi:10.1186/s12920-015-0147-4)
Supplement: Additional file 1: Figure S1. — Robustness of methylation subtypes. Principal component analysis to monitor data bias due to technical variables in the Bergen (A) and TCGA (B) cohorts, respectively. The entire set of 473,864 CpGs was used for principal component analysis. The heatmaps indicate the association of a sample annotation to each of the principal components. The strength of association is specified by the log10 p-value of the linear model with the respective principal component as dependent variable and sample annotation as regressor. DNAconc = DNA concentration. Technical variables of TCGA data are termed as defined by the TCGA consortium. TCGA = The Cancer Genome Atlas Consortium. Abbreviations as in Figure 1 of the main manuscript. (C) Sample overlap when using different CpG sets for group discovery in Bergen and TCGA data, respectively. For group discovery we used CpGs with variant methylation between melanoma and melanocytes. CpGs were required to be either methylated or de-methylated in at least 10 tumors for our final subtypes. The CpG set for 10 tumors contained 9,886 melanoma-methylated and 5,236 melanoma-demethylated CpGs. The heatmap displays the overlap of the resulting three consensus clusters at various tumor cutoffs. (D) Heatmap of TCGA sample co-occurrence between unsupervised consensus clusters and methylation subtypes obtained from (supervised) nearest centroid classification. Figure S2. Promoter island consensus clusters. The group discovery CpG set was reduced to CpGs located within 1500 bp upstream of transcription start sites and within a CpG island. In total this analysis includes 947 CpGs, of which 930 are hypermethylated, and 17 are hypomethylated in tumors as compared to melanocytes. Two-group consensus solutions were favorable to three-group solutions in Bergen and TCGA data, respectively. Figure S3. Signature expression across methylation subtypes in TCGA data. (A) ESTIMATE scores and tumor purity. P-value from Kruskal-Wallis test. (B) Mean expression values o [file 12920_2015_147_MOESM1_ESM.pdf]

Supplementary Figure 1

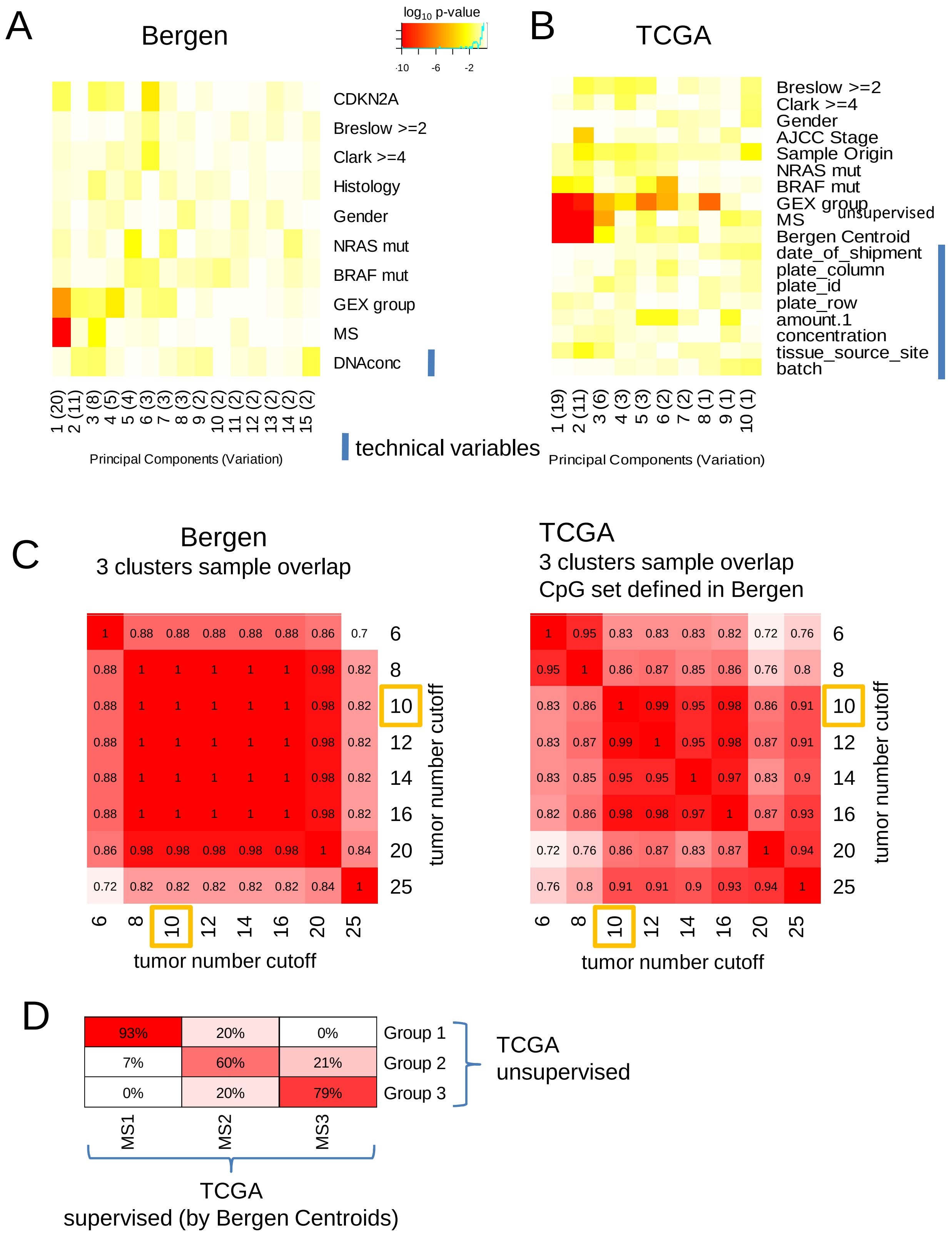

Supplementary Figure 2

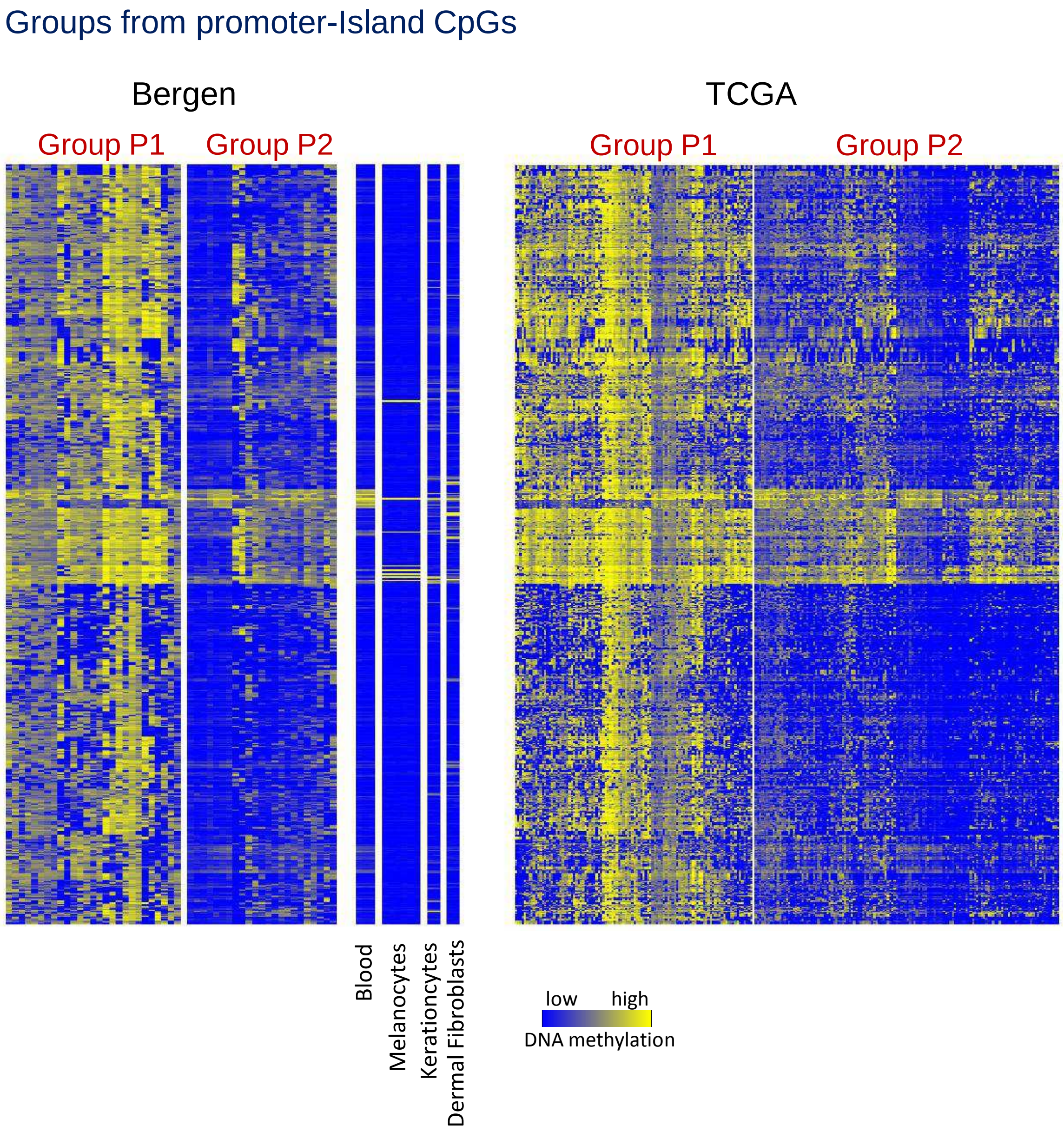

Supplementary Figure 3

A

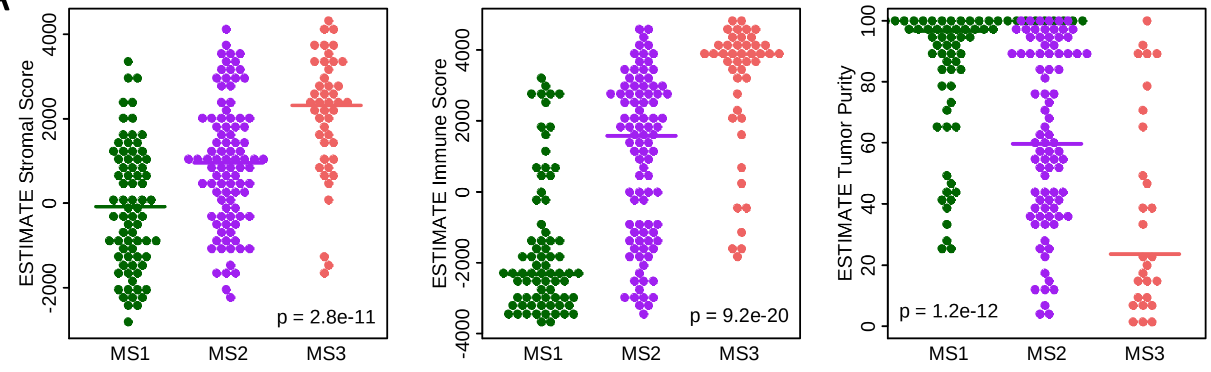

B

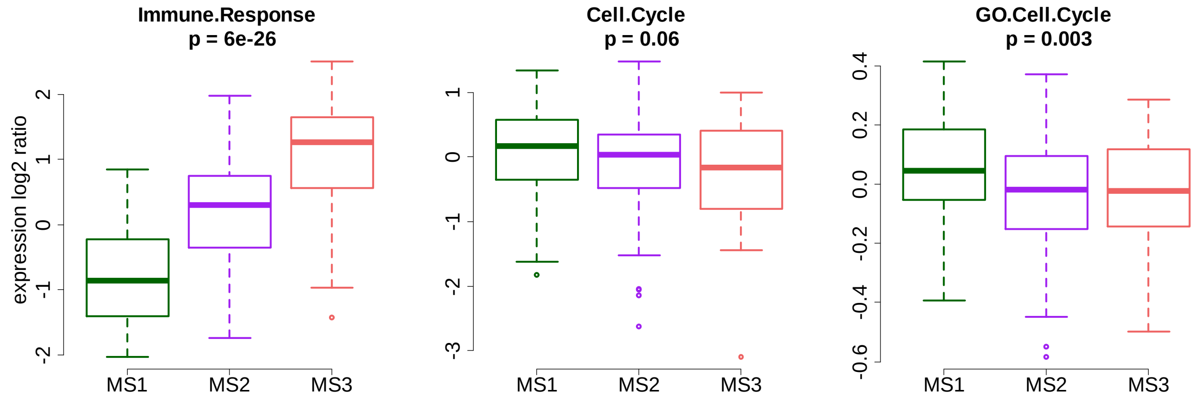

Supplementary Figure 4

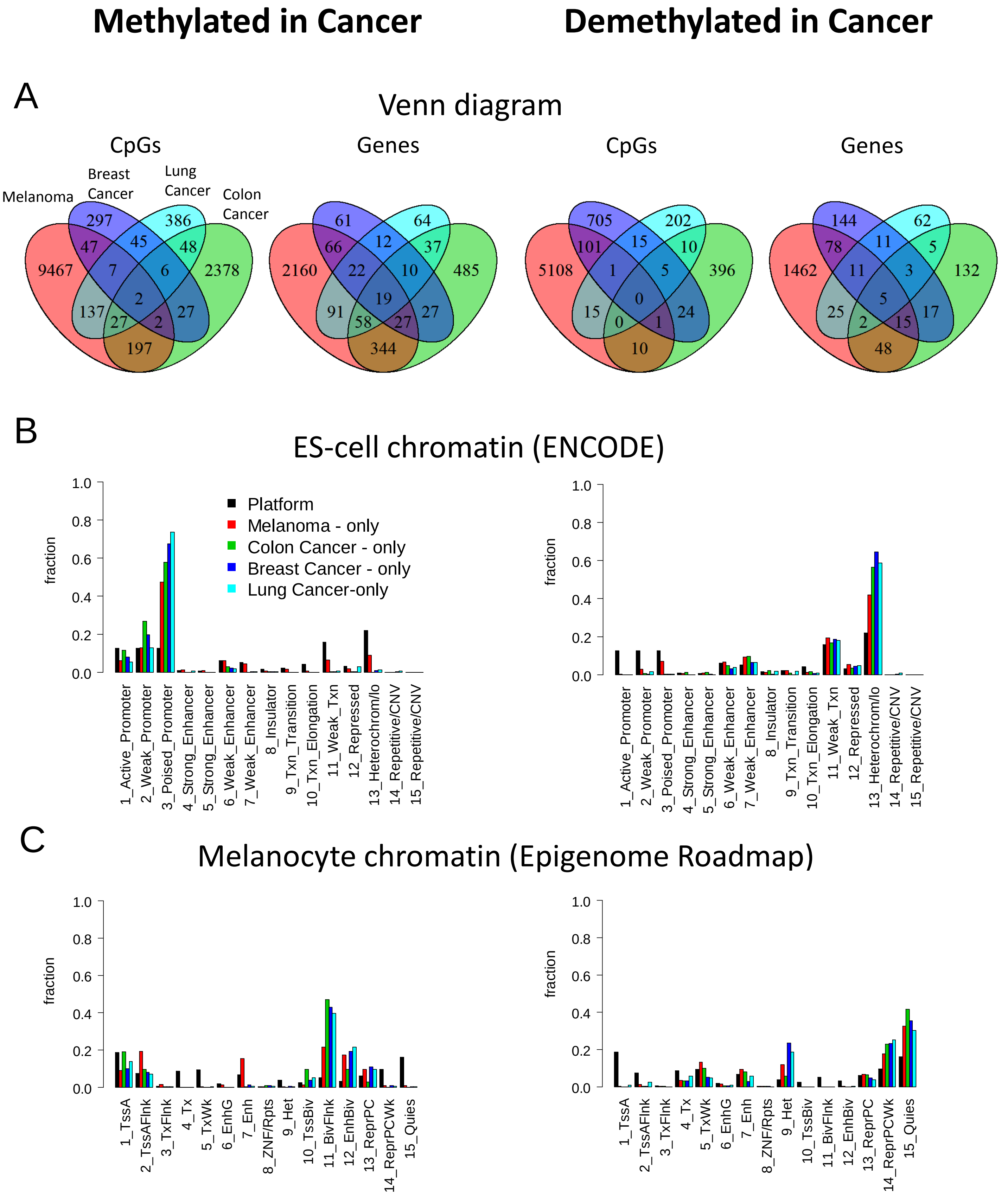

Supplementary Figure 5

A

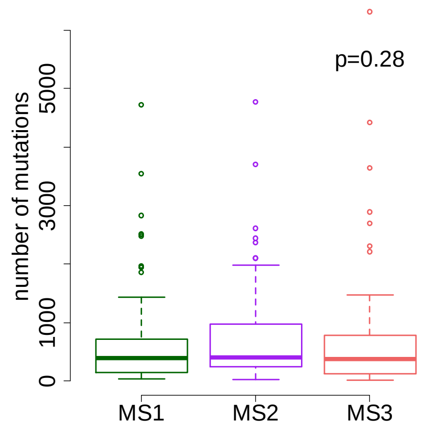

B

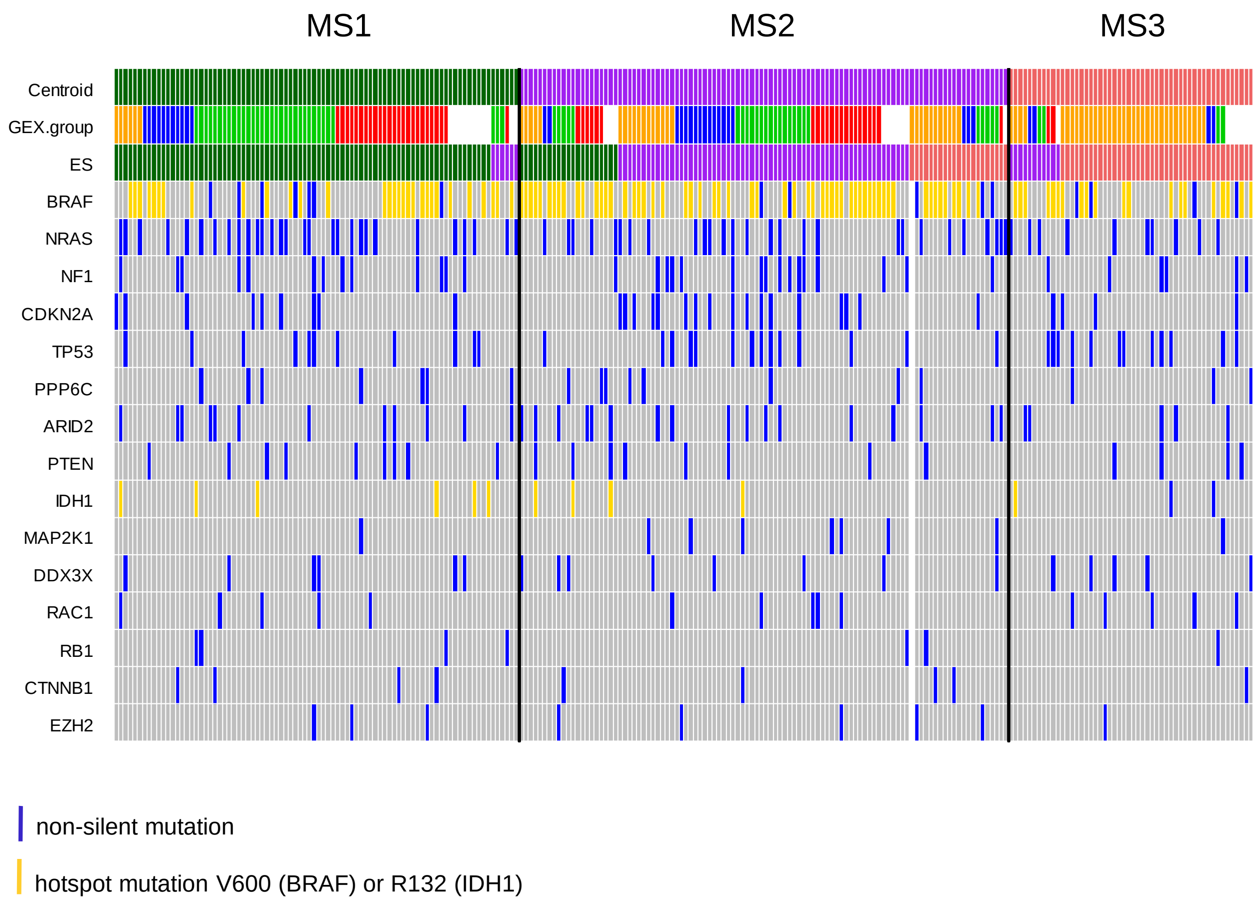

Supplementary Figure 6

A

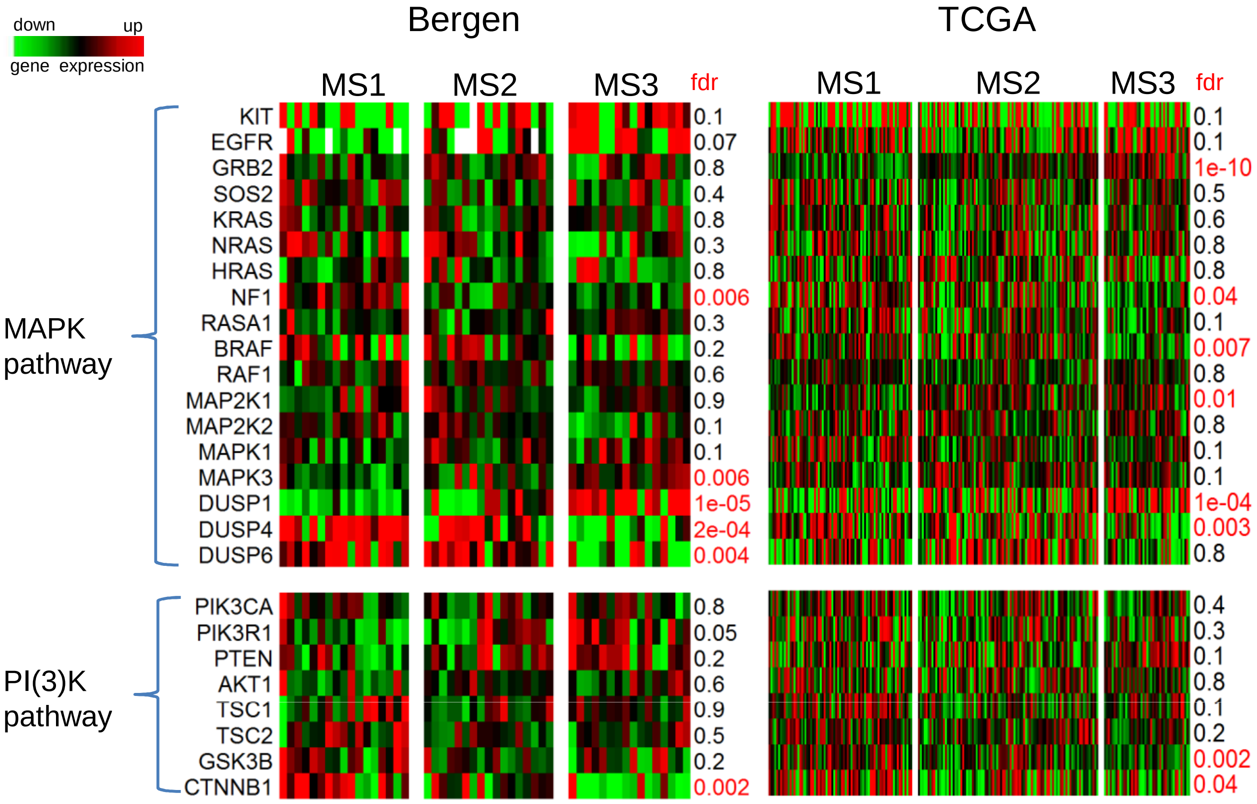

B

Bergen, $p = 0.0001$

	MS1	MS2	MS3
Immune-high	0	6	9
Normal-like	0	0	3
Pigmentation	9	8	3
Proliferative	8	3	1

TCGA, $p = 6 \times 10^{-14}$

	MS1	MS2	MS3
Immune-high	6	28	35
Normal-like	11	18	4
Pigmentation	33	26	4
Proliferative	25	22	2
